# Identification of Potential QTLs Related to Grain Size in Rice

**DOI:** 10.3390/plants12091766

**Published:** 2023-04-26

**Authors:** Jae-Ryoung Park, Jeonghwan Seo, Songhee Park, Mina Jin, O-Young Jeong, Hyun-Su Park

**Affiliations:** Crop Breeding Division, National Institute of Crop Science, Rural Development Administration, Wanju 55365, Republic of Korea; icd0192@korea.kr (J.-R.P.); rightseo@korea.kr (J.S.); thdgml157@korea.kr (S.P.); genemina@korea.kr (M.J.); joyoung@korea.kr (O.-Y.J.)

**Keywords:** rice, grain size, yield, breeding, QTL

## Abstract

Rice is a major crop, providing calories and food for most of the world’s population. Currently, the global population is rapidly increasing, and securing a yield of rice that can satisfy everyone is an ongoing challenge. The yield of rice can be increased by controlling 1000-grain weight as one of the important determining factors. Grain length, grain width, grain thickness, and 1000-grain weight, which determine grain size, are controlled by QTLs. To identify QTLs related to grain size, we screened and then mapped 88 RIL individuals derived from a cross between JJ625LG, which has a long grain size, long spindle-shaped grains, and low 1000-grain weight, and Namchan, which has short grains with round shape and heavy 1000-grain weight. In 2021 and 2022, 511 SNP markers were used to map QTLs related to grain size to a physical map. The QTLs found to be related to grain size are evenly distributed on chromosomes 2, 3, 5, 10, and 11. The mapping results also show that the QTLs *qGl3-2*, *qRlw3*, and *qRlw3-2* of chromosome 3, and qGt5 and *qRlw5* of chromosome 5 are, respectively, associated with *GS3* and *qSW5*, which are the major genes previously cloned and found to be related to grain size. In addition, *qGw10* and *qGw10-1*, which were additionally detected in this study, were found to be associated with *Os10g0525200* (*OsCPq10*), a potential candidate gene involved in controlling grain size. This gene codes for a cytochrome P450 family protein and is reported to have a positive effect on grain size by interacting with proteins related to mechanisms determining grain size. In particular, *OsCPq10* was screened in the same identified QTL region for 2 consecutive years, which is expected to have a positive effect on grain size. These results will be helpful for breeding elite rice cultivars with high yields through additional fine mapping related to grain size.

## 1. Introduction

Globally, food security is under threat because of continued population growth, decreases in agricultural land area, and the unpredictability of climate change, resulting in its emergence as a serious social problem [1]. However, over the past half-century, the Green Revolution has resulted in greatly improved rice yields and is gradually solving the problem of starvation [2,3]. However, recent and rapid climate change has resulted in decreased farmland area, resulting in stagnating or collapsing yields of the world’s major food crops, such as rice, corn, and wheat, with no signs that the situation will improve [4]. In order to feed the ever-growing world population, a continual increase in the yield of major food crops, including rice, is required [5].

Rice is one of the representative food crops sold to consumers in grain form, and the shape of the grain is directly related to appearance and quality, and thus closely associated with the commodity’s value [6,7]. The characteristics of rice grains determine their marketability and are determined by the complex action of factors such as chalkiness, transparency, and 1000-grain weight along with grain shape characteristics such as length, width, and thickness [8,9]. In particular, grain shape is determined by the ratio of grain width to grain length, which is recognized as a major factor in determining yield because it is very highly correlated with 1000-grain weight in addition to being an important trait involved in determining appearance [10,11]. In addition, because it is strongly related to yield, grain shape is one of the most frequently selected traits in the rice breeding process and can provide an excellent model for understanding rice breeding pedigrees [12].

In general, grain size and shape are regulated by complex interactions between major and minor QTLs and are important factors that determine yield and grain quality [13,14]. Therefore, numerous studies have been conducted to genetically map the QTLs associated with grain shape in rice. As a result, over the past few decades, many thousands of QTLs have been sought after in various mapping populations [10,15,16,17]. In addition, the function of some of these QTLs has been characterized at the genetic level [18,19]. *GS3* is the first gene reported to be related to grain shape, and it was demonstrated to increase grain length while regulating transmembrane proteins [20,21]. It is also known that activation of *DEP1* shortens the grain length [22]. *qGL3.1* controls panicle phosphorylation to determine the grain length and 1000-grain weight [23]. Moreover, *GW2*, identified on chromosome 2, is related to the grain width [24]. Loss of *GW2* function reportedly results in an increase in the number of cells and, consequently, in the grain width and 1000-grain weight [25]. The loss of function of *qSW5*/*GW5*, on chromosome 5, is related to the grain width and increases the yield by increasing the sink size [11]. *qSW5*/*GW5* interacts with calmodulin protein to regulate plasma-membrane-associated protein to narrow the grain width, and when its function is lost, the grain width and 1000-grain weight increase [11]. It has been reported that activation of *GS5*, located on chromosome 5, is involved in increasing grain width and 1000-grain weight [26]. In addition, in rice, *GW8*/*OsSPL16* [27], *GW7* [28], *GLW7* [29], *LGY3* [30], and *GS2* [31] were studied at the gene level and found to influence grain size and 1000-grain weight.

Herein, using a population derived from a cross between ‘JJ625LG’ and ‘Namchan’, we report on the identification of a dominant QTL related to grain shape that ultimately controls 1000-grain weight. The results of this study will be helpful for applying QTLs of the factors related to grain shape in the future, namely as cloning and breeding materials.

## 2. Results

### 2.1. Evaluation of Grain Size Phenotypes in the Mapping Population

**Figure 1:**
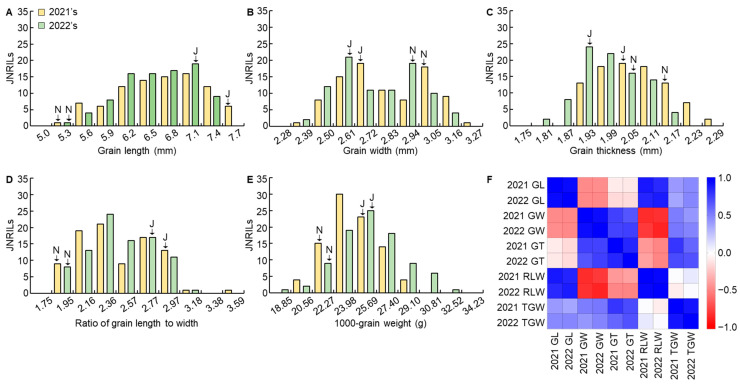
Histogram and correlation analysis of the traits related to grain size in JJ625LG/Namchan recombinant inbred lines (JNRIL). The frequency distributions for grain length (**A**), grain width (**B**), grain thickness (**C**), the ratio of grain length to width (**D**), and 1000-grain weight (**E**) related to grain size were analyzed. These were continually investigated in 2021 and 2022, and the investigated traits were evaluated to be normally distributed for both years. In addition, among the traits related to grain size, grain length and the ratio of grain length to width showed a strong positive correlation with each other and a weak positive correlation with 1000-grain weight (**F**). The correlation between grain width and grain thickness was strongly positive. J, JJ625LG; N, Namchan; GL, grain length; GW, grain width; GT, grain thickness; RLW, the ratio of grain length to width; TGW, 1000-grain weight.

To map the QTLs related to grain size, grain length, grain width, grain thickness, the ratio of grain length to width, and 1000-grain weight were investigated in JJ625LG, Namchan, and JNRILs (Table S1). JJ625LG had higher values than Namchan for grain length, the ratio of grain length to width, and 1000-grain weight. The grain width and grain thickness were higher in value for Namchan than JJ625LG. JJ625LG had long grains with a long spindle shape, and Namchan had short grains with a round shape. In addition, the traits related to the grain size of JNRILs were continuous and normally distributed, indicating that they were suitable for QTL mapping (Figure 1A–E). The traits related to grain size showed significant differences between the years of the study, 2021 and 2022, and the frequency distribution trends were similar. Among the traits related to grain size, the grain length and ratio of grain length to width are strongly positively correlated, and the grain width and thickness are also strongly positively correlated with each other (Figure 1F). However, the grain length, ratio of grain length to width, grain width, and grain thickness are negatively correlated with each other. We found that 1000-grain weight is positively correlated with the grain length and ratio of grain length to width.

### 2.2. Construction of a Physical Map and Mapping of QTLs Related to Grain Size

The QTLs related to grain size that were identified by applying a set of SNP markers were mapped to the mapped JNRIL populations. Of the 624 detected SNP markers, 511 SNP markers are polymorphic for JJ625LG and Namchan and were utilized for the analysis of QTLs related to grain size. These 511 SNP markers were found to be evenly distributed on 12 rice chromosomes. The total length of the constructed JNRIL linkage map is 1841.34 cm, and the average SNP interval is 3.93 cm. QTLs were mapped with the values of grain length, grain width, grain thickness, the ratio of grain length to width, and 1000-grain weight, and QTLs were detected by applying inclusive composite interval mapping (ICIM) and an empirical threshold with an LOD score of 3.0 or higher.

**Figure 2:**
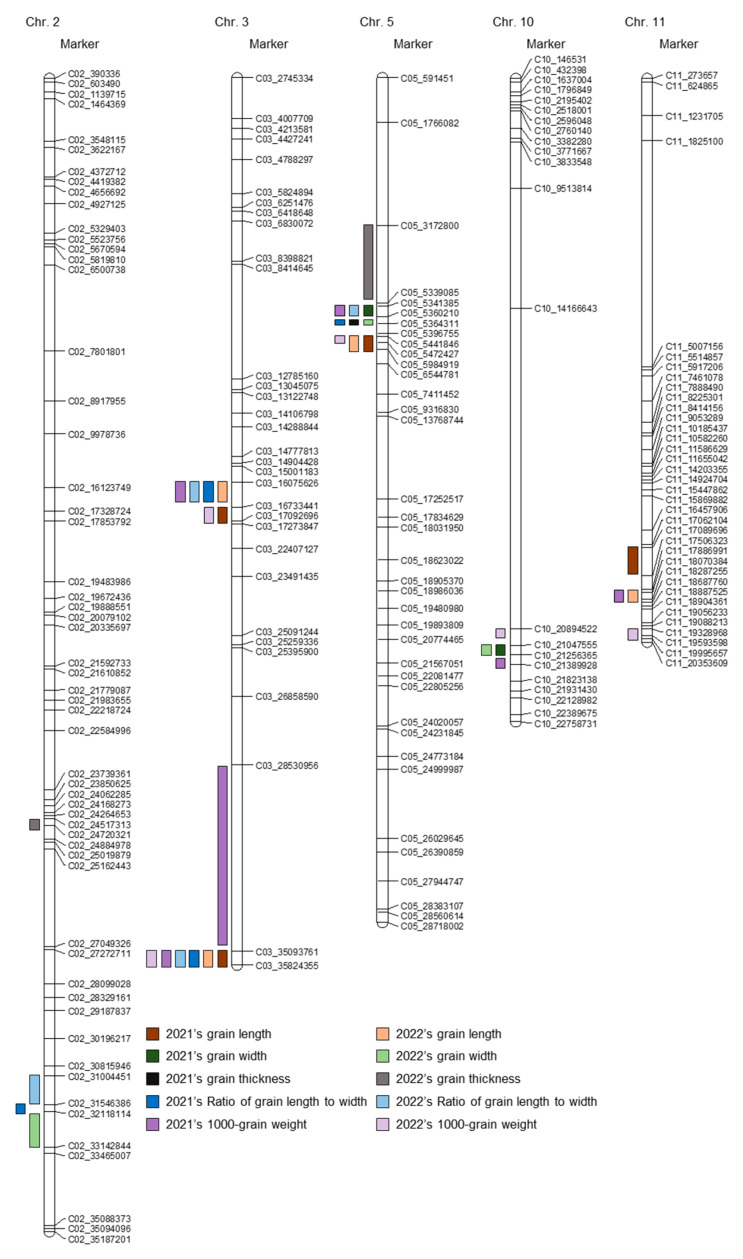
Construction of the JNRIL linkage map and QTL map of the traits related to grain size. A linkage map was constructed using 511 SNP markers. QTL were detected by applying the data of grain length, grain width, grain thickness, the ratio of grain length to width, and 1000-grain weight investigated in 2021 and 2022 to the JNRIL linkage map.

Regarding grain length, the maps produced for data from the two consecutive study years of 2021 and 2022 were identical for the region spanning C03_35093761–C03_35824355, which corresponds to the locations of *qGl3-1* and *qGl3-3*, respectively (Figure 2 and Table S2). The LOD scores of *qGl3-1* and *qGl3-3* were 10.9 and 14.2, and the explainable phenotypic variation was 13.9% and 20.0%, respectively. Moreover, also with respect to grain length, *qGl5* and *qGl5-1* were mapped to C05_5441846–C05_5472427 of chromosome 5, respectively, for both years. The LOD scores of *qGl5* and *qGl5-1* were 6.1 and 6.0, respectively, and the explainable phenotypic variation was 7.0% and 7.0%, respectively. Considering grain width, *qGw10* and *qGw10-1* were mapped to C10_21047555–C10_21256365 of chromosome 10, respectively, for both years. The LOD scores of *qGw10* and *qGw10-1* were 3.5 and 3.3, respectively, and the explainable phenotypic variations were 4.8% and 2.4%. For the ratio of grain length to width, *qRlw3* and *qRlw3-2* were mapped to C03_16075626–C03_16733441, and *qRlw3-1* and *qRlw3-3* to C03_35093761–C03_35824355 of chromosome 3, respectively, for both years. The LOD scores of *qRlw3* and *qRlw3-2* were 21.8 and 20.8, respectively, and the explainable phenotypic variation was 39.9% and 27.5%. The LOD scores of *qRlw3-1* and *qRlw3-3* were 4.4 and 8.1, respectively, and the explainable phenotypic variation was 4.8% and 7.2%. Regarding 1000-grain weight, *qTgw3-1* and *qTgw3-4* were mapped to C03_35093761–C03_35824355 of chromosome 3, respectively, for both years. The LOD scores of *qTgw3-1* and *qTgw3-4* were 3.3 and 6.2, respectively, and the explainable phenotypic variation was 5.2% and 6.7%. Among the QTLs consecutively mapped for both years, *qGw10* is an allele derived from Namchan, and the rest are alleles derived from JJ625LG.

### 2.3. Analysis of Genotypes Related to the Positive Effects of Grain Size

**Figure 3:**
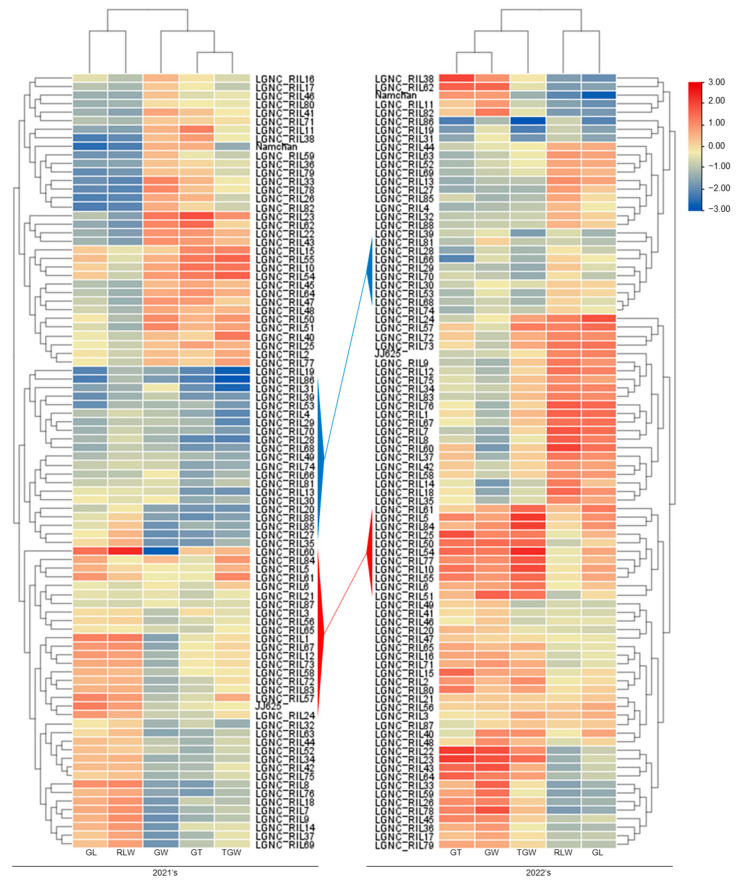
Group clustering using the correlation of traits related to grain size in the JNRILs. When the JNRILs were clustered using the correlation coefficients of grain length, grain width, grain thickness, the ratio of grain length to width, and 1000-grain weight investigated in 2021 and 2022, each group was classified. For two consecutive years, the lines clustered into a heavy group with long grains, a long spindle shape, and high 1000-grain weight (indicated by a red line, LLH group), and a group with short grains, a round shape, and a light 1000-grain weight (indicated by blue lines, SSL group).

In clustering analysis, the characteristics of JNRILs related to grain size, grain length, and the ratio of grain length to width were found clustered together, and 1000-grain weight, grain thickness, and grain width clustered together. In addition, through the use of the correlation coefficient, a pattern that was similarly separated by hierarchical clustering was visualized and is displayed in Figure 3. The JNRILs were clustered into various groups, and for two consecutive years, a line characterized by a long grain with a long spindle shape, and heavy 1000-grain weight and a line characterized by a short grain with a round shape and low 1000-grain weight were selected. When clustering was done for 2 years, we focused on lines that were grouped similarly. When the JNRILs were clustered using characteristics related to grain size, two groups were identified: the LLH group, with long grains, a long spindle shape, and a high 1000-grain weight, and the SSL group, with short grains, a round shape, and a low 1000-grain weight. Based on the investigation of grain characteristics in 2021 and 2022, LGNC_RIL5, LGNC_RIL6, LGNC_RIL61, and LGNC_RIL84 were included in the LLH Ip and LGNC_RIL29, LGNC_RIL30, LGNC_RIL39, LGNC_RIL53, LGNC_RIL66, LGNC_RIL68, LGNC_RIL70, LGNC_RIL74, and LGNC_RIL81 in the SSL group.

### 2.4. Screening of Candidate Genes Related to Grain Size

**Figure 4:**
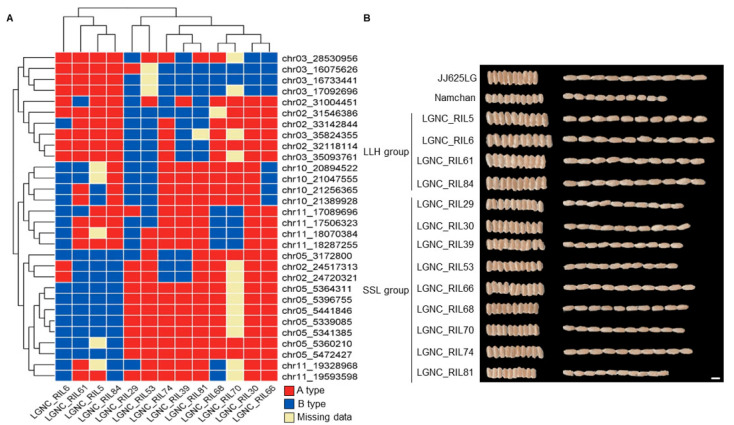
Analysis of the genotypic differences in the QTL regions of the LLH group and the SSL group in JNRIL. Among the marker regions detected simultaneously in 2021 and 2022 in the LLH group and SSL group, regions with different haplotypes were analyzed (**A**). Groups were clustered according to the type of marker region in the LLH group and SSL group. The morphological characteristics of rough and brown rice of the LLH group and SSL group are shown (**B**). The LLH group had greater grain length, width, and 1000-grain weight than the SSL group in brown rice.

**Figure 5:**
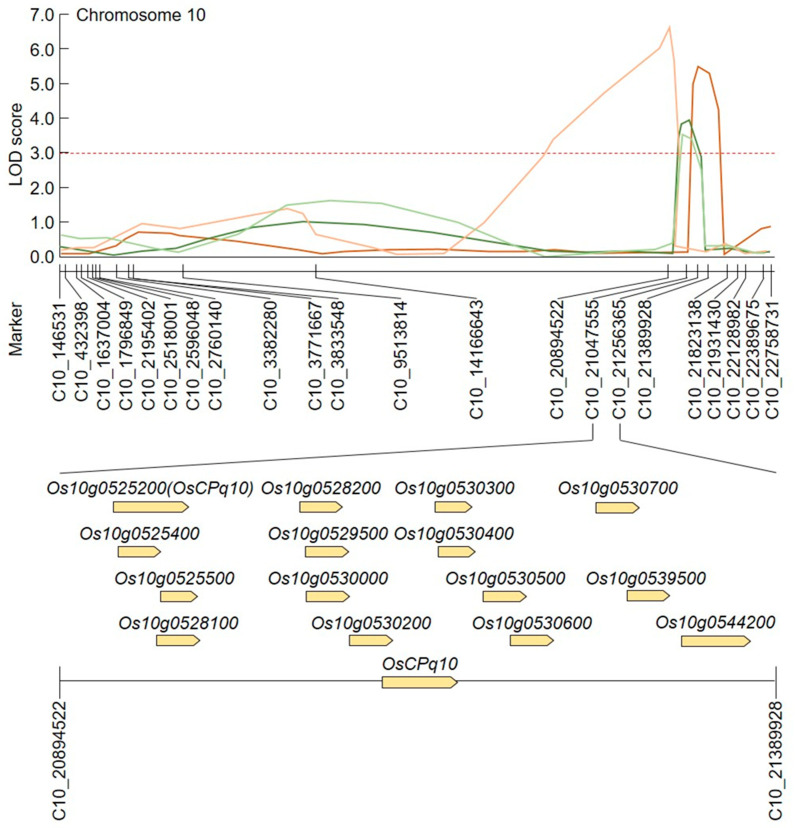
Construction of the physical map related to grain size in the JNRILs. A physical map for C10_146531–C10_22758731 of chromosome 10 was constructed. C10_21047555–C10_21256365 of chromosome 10 is a region in which the grain width and 1000-grain weight were detected with an LOD score of 3.0 or higher for 2 consecutive years. In this area, candidate genes that can affect grain size were screened. The size of the yellow box represents the relative size of the gene. And these are shown according to their relative position to C10_20894522-C10_21389928. Letters are arranged on different lines to avoid overlapping.

Candidate genes related to grain size were screened by focusing on the regions of mapped QTLs and, furthermore, those for which the maps were identical for both study years. In particular, we focused on regions where there were significant differences in the frequency of genotypes between the LLG and SSL groups (Figure 4 and Table S3), namely the regions C03_16075626, C03_16733441, C03_35093761, C03_35824355, C05_5360210, C05_5441846, C05_5472427, C10_21047555, and C10_21256365, within which we searched for candidate genes related to grain size. In the C03_35093761–C03_35824355 region, we identified *Os03g0397700*, encoding a protein similar to serine/threonine protein kinase-like protein; *Os03g0401100*, encoding a protein-kinase-domain-containing protein; *Os03g0401300*, encoding sucrose-UDP glucosyltransferase 2; *Os03g0836800*, encoding a protein similar to IAA-amino acid hydrolase 1; and *Os03g0836900*, encoding a protein similar to IAA-amino acid hydrolase 1, which all affect grain size. In C05_5441846–C05_5472427, we identified *Os05g0187100*, encoding a protein similar to hexokinase; *Os05g0187800*, encoding a protein similar to proline-rich protein 13; and *Os05g0188700,* encoding an N-terminal-domain-containing protein. Moreover, in C10_21047555–C10_21256365, we found *Os10g0525200*, encoding a cytochrome P450 family protein; *Os10g0525400*, encoding a protein similar to glutathione S-transferase GSTU31; *Os10g0525500*, encoding a protein similar to glutathione S-transferase GSTU31; *Os10g0528100*, encoding a protein similar to glutathione S-transferase GST 42; *Os10g0528200*, encoding a protein similar to glutathione S-transferase TSI-1; *Os10g0529500*, encoding a protein similar to glutathione S-transferase 2; *Os10g0530000*, encoding a protein similar to glutathione S-transferase GST 40; *Os10g0530200*, encoding a protein similar to glutathione S-transferase TSI-1; *Os10g0530300*, encoding a protein similar to glutathione S-transferase 2; *Os10g0530400*, encoding a protein similar to glutathione S-transferase Cla47; *Os10g0530500*, encoding a protein similar to glutathione g-transferase Cla47; *Os10g0530600*, encoding a protein similar to glutathione S-transferase GST 20; *Os10g0530700*, encoding a protein similar to glutathione S-transferase GST 38; *Os10g0530900*, encoding a protein similar to glutathione S-transferase GST 30; *Os10g0539500*, encoding histone H4; and *Os10g0544200*, encoding a basic helix–loop–helix dimerization region bHLH-domain-containing protein (Figure 5).

### 2.5. Analysis of the DNA and Protein Sequences of Candidate Genes Related to Grain Size

**Figure 6:**
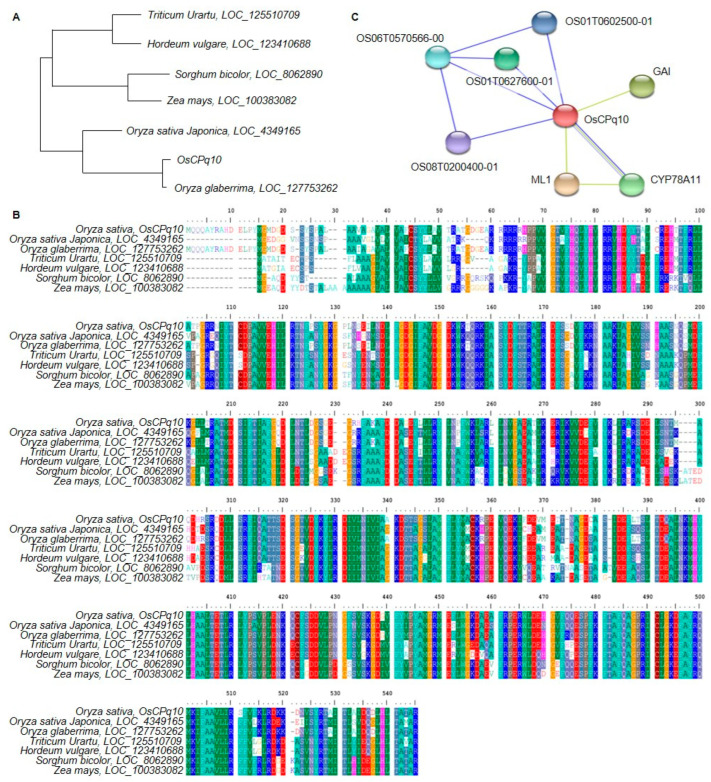
DNA and protein sequence analysis of *Os10g0525200*. *Os10g0525200* was revealed to have similar DNA (**A**) and protein (**B**) sequences to cytochrome P450 proteins from *Oryza sativa*, *Triticum*, *Hordeum*, *Zea mays*, and *Sorghum*. In addition, *Os10g0525200* was identified to have a high rate of homology with *Oryza sativa*, *Triticum*, *Hordeum*, *Zea mays*, and *Sorghum*. When the protein interactions of *Os10g0525200* were predicted, it interacted with proteins that positively affect grain size (**C**).

The DNA and protein sequences of candidate genes related to grain size were analyzed (Figure 6). Sequence analysis was performed using NCBI BLAST. In the C10_21047555–C10_21256365 region, which corresponds to chromosome 10, *Os10g0525200* (*OsCPq10*) was identified, which encodes a cytochrome P450 family protein. In addition, genetic similarities with DNA sequences coding for cytochrome P450 of *Oryza sativa japonica*, *Oryza glaberrima*, *Triticum urartu*, *Hordeum vulgare* subsp. *vulgare*, *Zea mays*, and *Sorghum bicolor* were identified in the phylogenetic tree. Among them, *OsCPq10* was most similar to the DNA sequence of cytochrome P450 (identity, 99%; similarity, 98.8%) in *Oryza glaberrima* and was most different from the DNA sequence of cytochrome P450 (identity, 88.0%; similarity, 80.6%) in *Triticum Urartu*. In addition, *OsCPq10* has a protein sequence similar to cytochrome P450 of *Oryza sativa japonica*, *Oryza glaberrima*, *Triticum urartu*, *Hordeum vulgare* subsp. *vulgare*, *Zea mays*, and *Sorghum bicolor* and has very high homology. *OsCPq10* interacts with OS01T0602500-01, a cytochrome-P450-like protein; OS06T0570566-00, an oxidoreductase protein; OS01T0627600-01, an oxidoreductase activity protein; OS05T0200400-01, which belongs to the cytochrome P450 family of proteins; GAI; DELLA protein SLR1, a probable transcriptional regulator that acts as a repressor of the gibberellin signaling pathway; ML1, a terminal ear1 protein homolog; a probable RNA-binding protein involved in the regular timing of lateral organ formation; and CYP78A11 cytochrome P450, which is involved in the regular timing of lateral organ formation, and all of them affect the grain size of rice.

## 3. Discussion

The rapid increase in the world population and the unpredictability of climate change have become important reasons to increase the yield of rice crops. To address this, the QTLs of traits related to yield were identified and mapped for use in actual field breeding. Grain size is directly related to yield [32]. Recently, as interest in grain quality has increased along with rapid population growth, there have been many attempts to search for genes that positively affect grain size and apply them to breeding programs [33]. In accordance with rapid climate change, the area corresponding to rice cultivation is decreasing [34]. Accordingly, it is necessary to screen candidate genes that have the potential to produce high yields in a limited area and apply them in breeding rice [34].

High yield can be achieved by adjusting the tiller number, panicle length, spikelet number, etc., but grain size is also an important factor for achieving high yield [35]. Much research has been conducted to increase yield by controlling grain size, and most of the currently identified QTLs are distributed in similar chromosomal regions [19,36,37,38]. Most of the QTLs related to grain size identified in this study were mapped to regions near previously reported QTLs. In particular, *GS3* and *qSW5* were identified in the QTLs detected on chromosomes 3 and 5, respectively, validating the results of this study and indicating the successful mapping of grain size QTLs using JNRILs [11,21]. Moreover, Hu et al. 2015 cloned *GS2* on chromosome 2, which was found to have a positive effect on grain size and yield [31]. *GS2* is found in the C02_28329161–C02_29187837 region of chromosome 2. However, in this study, a QTL was mapped in C02_31004451–C02_33142844 located downstream from C02_28329161–C02_29187837. The reason QTLs were mapped in different regions from those previously reported is that they could be detected in different regions depending on the breeding resources used and the environment in each study [39]. However, *GS3*, which was cloned as a major gene related to grain size and used as a breeding resource, was identified in C03_16075626–C03_16733441, identical to the results in Fan et al. [40], and *qSW5* was identified in C05_5360210–C05_5364311, identical to the results obtained by Liu et al. [11].

In this study, we focused on the C10_20894522–C10_21047555 region of chromosome 10, which was detected in addition to the previously reported region where major genes related to grain size were identified. Candidate genes that can affect grain size were searched for in the C10_20894522–C10_21047555 region, and most were found to encode cytochrome P450 proteins, glutathione S-transferase, histone proteins, and helix–loop–helix proteins [41,42,43,44]. All of these proteins are transcription factors that can control gene expression in rice, and the accumulation of genes with similar functions in the same region indicates the potential to act strongly on the expression of phenotypically expressed genes [45,46]. Here, the LLH and SSL groups were identified from the JNRILs for the two consecutive years of the study. These groups were compared, focusing on markers with different genotypes in the QTL region of chromosome 10 detected in this study. In addition, the genotypes of C10_21047555 and C10_21256365 differed between the two groups, due to which grain size and yield contrasted sharply. In this area, we screened for potential candidate genes related to grain size and finally selected *OsCPq10*. *OsCPq10* is predicted to encode cytochrome P450 and interact with proteins regulating grain size [47,48,49]. In particular, the increased activity of cytochrome P450 is reported to successfully lengthen grain size via effects on embryo development in rice [50]. In addition, it has been reported that the cytochrome P450 protein acts positively on high yield by increasing the grain weight of crops in which grain size plays an important role in yield, such as wheat [51], *Zea mays* [52], and barley [53], in addition to rice, which are the major grain species; cytochrome P450 protein has been shown to increase yield by positively controlling grain size. The set of SNP markers detected in this study was found to be evenly distributed throughout the rice genome and indicates the efficient identification of QTLs related to grain size. Nevertheless, the identified SNP markers did not cover some regions in some chromosomes, and the cause of this phenomenon was judged to be the differences between JJ625LG and Namchan, which are typical of *indica* and *japonica* [54]. In addition to this study, a similar phenomenon generally occurred when genetic maps or linkage maps were constructed using *japonica* and *indica* [55]. In order to solve this problem, it is necessary to compare the detailed genome sequences through the rearrangement of the entire genomes of JJ625LG and Namchan and to identify and discover additional markers. Nevertheless, in this study, both *GS3* and *qGW5*, which are the major genes regulating grain size, were identified in screening, as well as *OsCPq10*, which may act positively on grain size.

To continually provide calories to a growing population and respond to unpredictable climate change, it is necessary to screen and characterize additional QTLs related to grain size. In this study, candidate genes with the potential to positively control grain size for improving yield were found through screening. In addition, gene cloning and breeding of genome-edited plants using CRISPR/Cas9 is considered necessary for a complete understanding of gene function.

## 4. Materials and Methods

### 4.1. Plant Materials and Field Design

To map QTLs related to grain size in rice, we crossed ‘JJ625LG’, which has long grains with a long spindle shape and low 1000-grain weight, as the male parent, and ‘Namchan’, which has short grains with a round shape and heavy 1000-grain weight, as the maternal parent, and the mapping group of JJ625LG/Namchan recombinant inbred lines (JNRILs) were bred. The seeds of ‘JJ625LG’ and ‘Namchan’ were obtained from Dr. Hyun-Su Park of the Crop Breeding Division, National Institute of Crop Science (NICS), Rural Development Administration (RDA).

F_1_ seeds were derived by crossing ‘Namchan’ and ‘JJ625LG ’. After advanced generation in the field, 88 F_6_ individuals were transplanted to the field and used for genetic analysis. Mapping populations were transplanted into the fields of the NICS, RDA, in 2021 and 2022. While this study was in progress, the mapping population was bred and cultivated while complying with the international guidelines and legislation provided by the RDA in Korea [56]. The rice was bred and cultivated according to general local practices, which were implemented in compliance with the Convention on the Trade in Endangered Species of Wild Fauna and Flora (https://www.cites.org/ (accessed on 13 October 2020)). Before sowing, all seeds were sterilized in Spotak pesticide (25% Prochloraz, HANKOOKSAMGONG, Seoul, Republic of Korea) for 3 days in the dark at 33 °C. The sterilized seeds were sown on 13 April 2021 and 15 April 2022, and 30 days after sowing, transplanted to the field with 1 row for each line. Twenty-five plants were transplanted to 1 row, and the distance between the plants was 30 cm × 15 cm. In the field, standard fertilization (N–P_2_O_5_–K_2_O, 9–4.5–5.7 kg/10 ha) was applied. Nitrogen was divided into basal/tillering/panicle fertilizer at a ratio of 5:2:3; phosphoric acid was provided for a single application as basal fertilizer; and potassium was divided into basal/panicle fertilizer at a ratio of 7:3. Cultivation was carried out according to the standard method presented by the RDA [57].

### 4.2. Investigation of Traits Related to Grain Size

In order to investigate the grain characteristics of the mapping population, 10 plants for each line of JJ625LG, Namchan, and JNRILs were randomly harvested in bulk 40 days after heading. Grain size and 1000-grain weight were evaluated for brown rice using harvested seeds. Five characteristics were investigated, namely, grain length (GL, mm), grain width (GW, mm), the ratio of grain length to width (RLW), grain thickness (GT, mm), and 1000-grain weight (TGW, g). Characteristics related to grain size were investigated on 20 grains at random using an electronic digital caliper (Caliper CD-15CP, Mitutoyo Corp., Japan) with a precision of 0.001 mm. Grains of brown rice measuring 7.1 mm or more in length were classified as extra-long, 6.01–7.09 mm were classified as long, 5.51–6.00 mm were classified as medium, 5.01–5.50 mm were classified as medium–short, and shorter than 5.0 mm were classified as short. For the RLW, a score of 3.0 or more was classified as long spindle-shaped, 2.5–2.99 as spindle-shaped, 2.0–2.49 as half spindle-shaped, 1.5–1.99 as semi-round, and less than 1.5 as round [58]. TGW (g) was calculated as the weight of total grain harvested per plant divided by the number of spikelets multiplied by 1000. Correlation coefficients were calculated for GL, GW, GT, RLW, and TGW in JNRILs, and the latter were clustered by hierarchical clustering using the calculated correlation coefficients.

### 4.3. Extraction of Genomic DNA

Genomic DNA was extracted from JJ625LG, Namchan, and the JNRILs using the BioSprint 96 DNA Plant Kit (INDICAL BIOSCIENCE, Cat. SP947057, Leipzig, Germany). Leaf samples were ground after freezing in liquid nitrogen using a TissueLyser (Qiagen, Cat. 85300, Hilden, Germany), and genomic DNA was extracted in accordance with the manufacturer’s manual provided with the DNA extraction kit. The extracted DNA was diluted to 10 ng/μL after quantification and quality confirmation using a NanoDrop ND 1000 spectrophotometer (ThermoFisher, Cat. ND 1000, Waltham, MA, USA). The extracted DNA was mixed with 6× loading dye (SIGMA, Cat. G2526-5ML, Saint Louis, MO, USA) and loaded onto a 0.8% agarose gel (SIGMA, Cat. A9539-25G, Saint Louis, MO, USA) containing EtBr (SIGMA, Cat. E1510, Saint Louis, MO, USA). After electrophoresis, the DNA was identified using a UV transilluminator (Bio-Rad, Cat. 170–8070, Hercules, CA, USA).

### 4.4. Construction of JNRIL Linkage Maps

To identify the QTLs of traits related to grain characteristics in the JNRILs, 624 SNPs were detected in JJ625LG and Namchan. To construct the physical map of the JNRILs, 30% of the missing markers among the 624 detected SNPs were removed, and 10 overlapping SNP markers were additionally removed. Finally, we constructed a physical map of the JNRILs using 511 SNP markers. Physical maps were constructed using MapChaart 2.32 software.

### 4.5. Mapping of QTLs Related to Grain Size

The QTLs were searched using the surveyed values of traits related to grain size. IciMapping version 4.0 [59], a QTL analysis program, was used to detect QTLs related to grain size. The functions of ‘By LOD’ and ‘By Input’ of the ICIM program were applied for grouping each marker, and the ‘Kosambi’ function was applied for mapping. The I value used as the threshold for the searched QTLs was that calculated by repeating the permutations at the *p* < 0.05 level 1000 times. Each QTL was named according to the method proposed by McCouch [60].

### 4.6. Statistical Analysis

R (version 4.1.3, The R Foundation for Statistical Computing) was used for the statistical analysis of the investigated traits. Descriptive statistics, such as the average of each trait, and comparisons between averages using Duncan’s multiple range test (DMRT) were performed using the Agricole package in the R package. The significance of the average values was tested for significant differences at the *p* < 0.05 level according to the *t*-test and DMRT. Pearson’s correlation coefficient was analyzed by applying the psych package to clarify the relevance of the investigated traits, and the corr.test was applied to compare three or more traits. Corrplot was applied to construct various graphs and heatmaps.

## 5. Conclusions

Grain size in rice is directly related to yield, on which it has a very strong influence. QTLs related to grain size were mapped using JNRILs derived from crossing JJ625LG and Namchan. A linkage map of 511 SNP markers was constructed. In the QTL map for the two consecutive study years, *GS3* and *qSW5*, reported to be the major grain size genes, were identified on chromosomes 3 and 5, respectively. The clustering of JNRIL grain characteristics resulted in two groups: the LLH group and the SSL group. In addition, regions of genotypes detected at different frequencies in the LLH group and the SSL group were analyzed, and the C10_21047555–C10_21256365 region was identified as being important for grain size. *OsCPq10* was found in C10_21047555-C10_21256365, which encodes for the cytochrome P450 protein. *OsCPq10* interacts with proteins regulating grain size, and its homology with other proteins in crops was analyzed using Gramene. *OsCPq10* has the potential to be used for breeding an elite rice cultivar capable of producing high yields by acting positively on grain size.

## Data Availability

The data presented in this study are available on request from the corresponding author.

## Supplementary Materials

The following supporting information can be downloaded at: https://www.mdpi.com/article/10.3390/plants12091766/s1, Table S1: Grain size related traits in JJ625/Namchan recombinant inbred lines (JNRIL). Table S2: Information of QTL related to grain size from JJ625/Namchan recombinant inbred lines. Table S3: Candidate genes related to grain size.
