# Supplementary material for: Identification of Potential QTLs Related to Grain Size in Rice
Source: Plants (Basel). 2023 Apr 26;12(9):1766. doi: 10.3390/plants12091766 (PMC10181466; doi:10.3390/plants12091766)
Supplement: Supplementary file 1 [file plants-12-01766-s001.zip › plants-2340305-supplementary.pdf]

Supplementary Materials

Table S1. Grain size related traits in JJ625/Namchan recombinant inbred lines (JNRIL).

Trait[z]	Year	Parents		JNRIL
		JJ625LG	Namchan	
GL (mm)	2021	7.64±0.13	5.25±0.07	6.55±0.60
	2022	7.06±0.06	5.06±0.09	6.47±0.49
GW (mm)	2021	2.70±0.09	2.99±0.08	2.77±0.21
	2022	2.58±0.06	2.88±0.13	2.72±0.20
GT (mm)	2021	2.05±0.06	2.14±0.04	2.04±0.09
	2022	1.91±0.06	2.05±0.09	1.97±0.08
RLW	2021	2.83±0.05	1.76±0.04	2.38±0.34
	2022	2.74±0.05	1.76±0.09	2.40±0.31
TGW (g)	2021	24.25±0.23	21.90±0.17	23.84±2.00
	2022	24.75±0.44	21.71±0.45	25.17±2.62

[z]GL, grain length; GW, grain width; GT, grain thickness; RLW, ratio of grain length to width; TGW, 1000-grain weight.

Table S2. Information of QTL related to grain size from JJ625/Namchan recombinant inbred lines.

Trait	Year	Chromosome	QTL	Marker interval	LOD[z]	PVE[y]	Add[x]	Increasing effects[w]
GL	2021	3	qGl3	chr03_16733441-chr03_17092696	26.7	55.7	0.5	JJ625LG
		3	qGl3-1	chr03_35093761-chr03_35824355	10.9	13.9	0.2	JJ625LG
		5	qGl5	chr05_5441846-chr05_5472427	6.1	7.0	0.2	JJ625LG
		11	qGl11	chr11_17089696-chr11_17506323	5.9	7.0	0.2	JJ625LG
	2022	3	qGl3-2	chr03_16075626-chr03_16733441	25.1	50.8	0.3	JJ625LG
		3	qGl3-3	chr03_35093761-chr03_35824355	14.2	20.0	0.2	JJ625LG
		5	qGl5-1	chr05_5441846-chr05_5472427	6.0	7.0	0.1	JJ625LG
		11	qGl11-1	chr11_18070384-chr11_18287255	5.7	6.7	0.1	JJ625LG
GW	2021	5	qGw5	chr05_5341385- chr05_5360210	25.5	65.9	0.0	JJ625LG
		10	qGw10	chr10_21047555-chr10_21256365	3.5	4.8	-0.1	Namchan
	2022	2	qGw2	chr02_32118114-chr02_33142844	4.9	4.0	0.0	Namchan
		5	qGw5-1	chr05_5360210-chr05_5364311	37.8	78.3	-0.2	Namchan
		10	qGw10-1	chr10_21047555-chr10_21256365	3.3	2.4	0.0	JJ625LG
GT	2021	5	qGt5	chr05_5360210-chr05_5364311	15.6	54.0	-0.1	Namchan
	2022	2	qGt2	chr02_24517313-chr02_24720321	4.7	7.0	0.0	Namchan
		5	qGt5-1	chr05_3172800-chr05_5339085	23.9	63.2	-0.1	Namchan
RLW	2021	2	qRlw2	chr02_31546386-chr02_32118114	3.6	3.8	0.1	JJ625LG
		3	qRlw3	chr03_16075626-chr03_16733441	21.8	39.9	0.2	JJ625LG
		3	qRlw3-1	chr03_35093761-chr03_35824355	4.4	4.8	0.1	JJ626LG
		5	qRlw5	chr05_5360210-chr05_5364311	20.8	36.7	0.2	JJ627LG
	2022	2	qRlw2-1	chr02_31004451-chr02_31546386	5.1	4.6	0.1	JJ628LG
		3	qRlw3-2	chr03_16075626-chr03_16733441	20.8	27.5	0.2	JJ629LG
		3	qRlw3-3	chr03_35093761-chr03_35824355	8.1	7.2	0.1	JJ630LG
		5	qRlw5-1	chr05_5341385-chr05_5360210	29.2	49.2	0.2	JJ631LG

TGW	2021	3	qTgw3	chr03_16075626-chr03_16733441	9.1	17.0	0.9	JJ632LG
		3	qTgw3-1	chr03_35093761-chr03_35824355	3.3	5.2	0.5	JJ633LG
		5	qTgw5	chr05_5341385-chr05_5360210	17.2	39.9	-1.3	Namchan
		10	qTgw10	chr10_21256365-chr10_21389928	5.0	8.2	0.6	JJ633LG
		11	qTgw11	chr11_18070384-chr11_18287255	2.6	4.1	0.4	JJ634LG
	2022	3	qTgw3-2	chr03_16733441-chr03_17092696	16.2	23.2	1.3	JJ635LG
		3	qTgw3-3	chr03_28530956-chr03_35093761	4.5	4.5	0.6	JJ636LG
		3	qTgw3-4	chr03_35093761-chr03_35824355	6.2	6.7	0.7	JJ637LG
		5	qTgw5-1	chr05_5396755-chr05_5441846	22.3	38.1	-1.6	Namchan
		10	qTgw10-1	chr10_20894522-chr10_21047555	6.6	7.4	0.7	JJ636LG
		11	qTgw11-1	chr11_19328968-chr11_19593598	3.6	3.6	0.5	JJ636LG

[z] Logarithm of the odds

[y] Phenotypic variation explained, a ratio that can explain the variation in grain traits in the JJ625LG/Namchan RIL

[x] Additive effect, Positive values of the additive effect indicate that alleles from JJ625LG are in the direction of increasing the traits

[w] Increase allele is the source of the allele causing an increase in the measured trait

Table S3. Candidate genes related to grain size.

Locus ID / Links	Accession	Description
Os10g0525200		Cytochrome P450 family protein.
Os10g0525400	AK063207	Similar to Glutathione S-transferase GSTU31 (Fragment).
	AF402802	Similar to Glutathione S-transferase GSTU31 (Fragment).
	AK104795	Similar to Glutathione S-transferase GSTU31 (Fragment).
	AK104194	Similar to Glutathione S-transferase GSTU31 (Fragment).
	AK067309	Similar to Glutathione S-transferase GSTU31 (Fragment).
Os10g0525500	AK063104	Similar to Glutathione S-transferase GSTU31 (Fragment).
Os10g0528100	AK108376	Similar to Glutathione S-transferase GST 42 (EC 2.5.1.18) (Fragment).
Os10g0528200	CI464636	Similar to Glutathione S-transferase TSI-1 (EC 2.5.1.18) (Glutathione S- transferase 1).
Os10g0529500	CI555732	Similar to Glutathione-S-transferase 2.
Os10g0530000	CI239374	Similar to Glutathione S-transferase GST 40 (EC 2.5.1.18).
Os10g0530200	AK107032	Similar to Glutathione S-transferase TSI-1 (EC 2.5.1.18) (Glutathione S- transferase 1).
Os10g0530300		Similar to Glutathione-S-transferase 2.
Os10g0530400	AK105926	Similar to Glutathione-S-transferase Cla47.
	AK067361	Similar to Glutathione-S-transferase Cla47.
	AK104213	Similar to Glutathione-S-transferase Cla47.
	AK098972	Similar to Glutathione-S-transferase Cla47.
Os10g0530500	AF402800	Similar to Glutathione-S-transferase Cla47.
Os10g0530600	AK109314	Similar to Glutathione S-transferase GST 20 (EC 2.5.1.18).
Os10g0530700		Similar to Glutathione S-transferase GST 38 (EC 2.5.1.18).
Os10g0539500	AK119699	histone H4 [Oryza sativa (japonica cultivar-group)].
	D10397	histone H4 [Oryza sativa (japonica cultivar-group)].
	AK059098	histone H4 [Oryza sativa (japonica cultivar-group)].
Os10g0544200	AK063669	Basic helix-loop-helix dimerisation region bHLH domain containing protein.
